# Inhibition of HIV-1 Replication by Balsamin, a Ribosome Inactivating Protein of *Momordica balsamina*

**DOI:** 10.1371/journal.pone.0073780

**Published:** 2013-09-05

**Authors:** Inderdeep Kaur, Munish Puri, Zahra Ahmed, Fabien P. Blanchet, Bastien Mangeat, Vincent Piguet

**Affiliations:** 1 Department of Microbiology and Molecular Medicine, University of Geneva, Switzerland University of Geneva, Geneva, Switzerland; 2 Fermentation and Protein Biotechnology Laboratory, Department of Biotechnology, Punjabi University, Patiala, India; 3 Department of Dermatology and Wound Healing, Institute of Infection and Immunity, Cardiff University School of Medicine, Cardiff, Wales, United Kingdom; Lady Davis Institute for Medical Research, Canada

## Abstract

Ribosome-inactivating proteins (RIPs) are endowed with several medicinal properties, including antiviral activity. We demonstrate here that the recently identified type I RIP from *Momordica balsamina* also possesses antiviral activity, as determined by viral growth curve assays and single-round infection experiments. Importantly, this activity is at play even as doses where the RIP has no cytotoxic effect. In addition, balsamin inhibits HIV-1 replication not only in T cell lines but also in human primary CD4^+^ T cells. This antiviral compound exerts its activity at a viral replicative step occurring later than reverse-transcription, most likely on viral protein translation, prior to viral budding and release. Finally, we demonstrate that balsamin antiviral activity is broad since it also impedes influenza virus replication. Altogether our results demonstrate that type I RIP can exert a potent anti-HIV-1 activity which paves the way for new therapeutic avenues for the treatment of viral infections.

## Introduction

Ribosome-inactivating proteins (RIPs) are RNA N-glycosidases which cleave N-glycosidic bond of adenine A2660 in *Escherichia coli* 23 S rRNA or A4324 in eukaryotic 28 S rRNA located in a highly conserved α-sarcin/ricin (SR) loop on the rRNA. This results in depurination of SR loop and inability of the ribosome to bind elongation factor 2 and thus inhibit protein synthesis [1]. RIPs are classified into three types: Type I RIP, which are single chain highly basic proteins of approximately 30 kDa and possess enzymatic activity; Type II RIP, which are heterodimeric proteins composed of an enzymatically active A chain of approximately 30 kDa and a lectin-like B-chain of approximately 35 kDa [2]; and type III RIPs, which consist of a single enzymatically active polypeptide that is synthesized as a zymogen [3]. Type II RIPs such as ricin are usually more toxic than type I RIPs [4]. Ribosome inactivating proteins (RIPs) have multiple biological properties comprising anti-tumor, antiviral, abortifacient, and immunosuppressive activities either alone or conjugated with antibody as immunotoxins [5]. RIPs-based immunotoxins have been prepared for antitumor [6] and antiviral therapy [7].

RIPs are found abundantly in the seeds of several plant families, amongst which Caryophyllaceae, Cucurbitaceae, Euphorbiaceae and Phytolaccaceae. Several RIPs have been purified and investigated for their potential medicinal usage, including *Momordica charantia*, *Gelonium multiflorum*, *Phytolacca americana*, *Trichosanthes kirilowii*, *Luffa cylindrica*, *Bryonia dioica*, *Dianthus caryophyllus*, *Ricinus communis* and *Abrus precatorius* [8]. Importantly, many type I and type II RIPs, amongst which α-and β-MMC, MAP30, GAP31, TAP29, DAP30, DAP32, TCS, PAP, bryodin and ricin, have been reported to inhibit HIV-1 replication *in vitro* and *in vivo* [9]. Nevertheless, the anti-HIV mechanism of ribosome inactivating protein is still not clear.

Extracts from *Momordica charantia,* which belongs to the Cucurbitaceae family, have been used as therapeutic agent for centuries. Accordingly, fruits and seeds extracts of this plant have been shown to possess *in vivo* anti-tumor activity, immune enhancement ability and effect on HIV-1 [10]. In recent years, several type I RIPs have been isolated from this edible plant, namely α-momorcharin, β-momorcharin, MAP30, γ-momorcharin, δ-momorcharin, ε-momorcharin and charantin [11]. While all of these RIPs are endowed with N-glycosidase activity, only MAP30, α-and β-momorcharins were shown to possess anti-HIV activity [4]. While alpha momorcharin inhibits HIV replication in both acutely infected lymphoblastoid cells and chronically infected macrophages [12], MAP30 has anti-tumor activity and inhibits HIV-1 infection in both T cells and macrophages [13].

*Momordica balsamina* (commonly known as Balsam apple, bitter melon), a high-climbing vine from family Cucurbitaceae, is native to the tropical regions of Africa, Arabia, Asia and Caribbean. This plant is a monoecious vine and found in North India [14]. While *M. balsamina* solvent extract has shown *in vitro* and *in vivo* anti-malarial activity [15], its fruit and leaves extract has anti-hypoglycemic effect on rats [16]. Balsamin is a type I ribosome inactivating protein of 28 kDa that has recently been isolated from the seeds of *Momordica balsamina*. It inhibits protein synthesis in cell free lysate and possesses N-glycosidase activity [17]. In the present study, the anti-HIV-1 activity of purified balsamin was investigated. We report the inhibition of HIV-1 replication by a series of assays in both the Jurkat T cell line and primary T cells. In addition, we demonstrate that this antiviral factor acts by inhibiting a late viral replicative step, most likely the viral protein translation. Finally, we establish that balsamin antiviral activity is broad since it also blocks influenza virus replication. These observations may open new therapeutic avenues for the treatment of viral infections.

## Methods

### Cells and Viruses

The Jurkat human T cell line was maintained in RPMI-1640 medium (Life Technologies) supplemented with 10% heat inactivated fetal calf serum (FCS), 100 U/ml penicillin, 100 µg/ml streptomycin and 2 mM L-glutamine. The epithelial cell lines MDCK [18] and A549 [19] from dog and human origin respectively, were cultured in Dulbecco’s modified Eagle’s medium (Invitrogen) with 10% heat inactivated FCS, penicillin and streptomycin and glutamine. Blood samples and cell protocols were approved by the ethical committee (“Commission d’éthique de la recherche sur l’être humain” of the University of Geneva (Switzerland). Written informed consent was provided by study participants and validated by the institutional review board. Primary CD4^+^ T cells were isolated from buffy coats of healthy seronegative blood donors. CD4^+^ T cells were purified from PBMCs after Ficoll gradient separation with CD4^+^ T cell isolation kit II (Miltenyi Biotec), in accordance with the manufacturer’s instructions and maintained in RPMI 1640. Later on CD4^+^ T cells were activated by PHA-L (1 µg/ml) and IL-2 (20 ng/ml) prior to infection.

HIV-1 stock (R9 strain) was initially produced by transient transfection of 293 T cells. For single-round infections, we used an HIV-1 deleted for the *env* gene and pseudotyped with the surface G protein of vesicular stomatitis virus (VSV). Influenza A/PR8/34 (H1N1) strain was produced by infection of MDCK cells at a moi of 0.001, followed by culture for 72 hours in serum-free Opti-MEM supplemented with 1 µg/ml TPCK-treated trypsin (Sigma).

### Source and Purification of Balsamin

Balsamin was purified from the seeds of *Momordica balsamina* as described previously [17].

### Protein Analysis

Cells were lysed with RIPA buffer. Resulting extract were then pre-cleared (10′000×g spin for 10 minutes), and their protein content was quantified with the BCA kit (Thermo). Subsequent Western blotting analyses were performed according to standard procedures. Antibodies serving for the detection of actin (Millipore) and M1 (clone GA2B, Abcam) were of mouse origin. Gag p55 and p24 were detected with the mouse monoclonal antibody made by Bruce Chesebro and Kathy Wehrly (obtained through the AIDS Research and Reference Reagent Program, Division of AIDS, NIAID, NIH) [20].

### HIV-1 Viral Particles Quantification

The production of HIV-1 viral particles was quantified by 2 methods, both on cell-free supernatant after filtration through 0.45 µm pore-size nitrocellulose membrane (Spin-X; Corning). Firstly, the RT assay measures the reverse transcriptase (RT) enzymatic activity in the cells supernatant and was performed according to standard protocol [21]. Secondly, the p24-specific ELISA assay, performed by HIV-1 p24^CA^ Antigen Capture Assay kit from AIDS & Cancer Virus Program, which measures the amount of viral capsid protein in the supernatant.

### T Cells Infections with Wild Type HIV-1

In the presence of indicated dose of balsamin, Jurkat T cells (7×10^5^ cells/ml) were infected with HIV-1 at a multiplicity of infection (moi) of 0.01. Eight hours post-infection, the cells were washed with PBS and incubated with or without balsamin in culture medium. At indicated days, an aliquot of cell-free supernatant for each sample was harvested for determination of p24 and RT assay.

Primary CD4^+^ T cells (7×10^5^ cells/ml) were infected with HIV-1 at 0.1 multiplicity of infection (moi). AZT (Azidothymidine) was included as positive control of inhibition and three different balsamin concentration (0.22, 1.12 and 3.57 µM) used. The wells without protein were used as a negative control. After 8 h of infection, cells were washed with PBS and resuspended in fresh RPMI medium and cultured in the presence and absence of balsamin. At indicated days post-infection cell-free supernatant was collected to determine RT assay.

### HIV-1 Single Round Infections

Primary CD4^+^ T cells (8×10^5^ cells/ml) were pretreated with indicated concentrations of balsamin and with AZT as a control. The cells without balsamin were included as a negative control. Cells were then infected with indicated doses of HIV-1 Δ*env* pseudotyped with VSV G protein. Eight hours post -infection cells were washed with PBS and cultured with different concentration of balsamin. Forty-eight hours of post-infection, supernatant was collected to determine reverse transcriptase activity assay. In parallel, cell lysates were prepared for Western blot analysis.

### Cellular Viability and Balsamin TC50 Determination

For cell viability, Jurkat cells or primary CD4^+^ T cells were seeded at a density of 7×10^5^ in a 12 well plates. Cells were then treated with different concentration of balsamin in duplicate and incubated at 37°C. The wells without balsamin were used as control. Cell suspension was collected on indicated days to assess cell viability by counting cells in presence of Trypan blue solution. To determine TC50 of balsamin, Jurkat or primary CD4^+^ T Cells at 1×10^5^ in duplicate were incubated in 96 well plates with Balsamin at 0.2 µM, 2 µM and 20 µM. Treatments with AZT were also done as a drug toxicity control (not shown). Cells were harvested two days later for determination of cell viability by trypan blue exclusion and cell counting. In parallel, the same methodology was done but cells were harvested, stained with PE Annexin V Apoptosis Detection Kit I (BD Pharmingen) and viable (Annexin V^−^/7-AAD^−^), early apoptotic (Annexin V^+^/7-AAD^−^) and late apoptotic/necrotic (Annexin V^+^/7-AAD^+^) were determined.

### Balsamin IC_50_ Measurement

Jurkat cells were seeded (7×10^5^ cells/ml) in 12 well plates. In the presence of different concentration of balsamin, cells were infected with HIV-1 at a multiplicity of infection (moi) of 0.1. Eight hours post-infection cells were washed with PBS to remove free virus and then again resuspended in RPMI fresh medium. On 3^rd^ day post-infection, cell-free supernatant was collected to perform a RT assay. The inhibition of HIV-1 replication by 50% (IC_50_) was determined by plotting the results of the dose-response in Excel, and fitting a logarithmic trendline to the curve. Using to the equation of this trendline allowed us to determine the balsamin dose at which 50% of HIV-1 production was inhibited.

### Determination of Drug-Resistant Escape Mutant

To examine whether drug-resistant escape mutants could be generated in the presence of sub-optimal doses of balsamin, Jurkat T cells (1×10^5^ cells/well) were left untreated or incubated with increasing concentrations of Balsamin (0.1 nM, 1 nM, 10 nM) for 3 hours. Cells were in parallel treated with AZT (50 µM). Cells were then infected with HIV-1 at a multiplicity of infection of 1.0. The day after viral challenge, cells were washed with PBS and re-supplied with drugs. Cell free supernatants were preserved for p24 determination by ELISA.

### PCR

Jurkat cell line (7×10^5^ cells/ml) was pretreated with 3.57 µM of balsamin. The cells without balsamin were included as control. HIV-1 was DNase-treated for 30 minutes at 37°C to avoid carry-over of the plasmids used for viral particles production. Jurkat cell line was then infected with this virus a multiplicity of infection (moi) of 0.2 and 1. Heat inactivated virus was used as a negative control. Eight hours post-infection, cells were washed with PBS and cultured with 3.57 µM of balsamin. Forty-eight hours of post-infection, total cell-associated DNA was isolated with DNeasy kit (Qiagen) and amplified by PCR using primers targeting so-called *late reverse transcripts* (forward: TGTGTGCCCGTCTGTTGTGT; reverse: GAGTCCTGCGTCGAGAGAGC) [22]. The amplification of actin with actin-specific primers (forward: TCACCCACACTGTGCCCATCTACGA; reverse: CAGCGGAACCGCTCATTGCCAATGG) served as an input control. In parallel, the supernatant was harvested to determine reverse transcriptase activity assay.

### Influenza virus Infection and Titration

A549 cells were split in 6 well plates and infected with influenza virus (PR8). The virus infected cells were incubated in the presence of different concentration of balsamin. The wells without protein served as a negative control. After 16 h of incubation, cells were twice washed with PBS and suspended in OPTI-MEM medium. Twenty four hours after the PBS wash, supernatant was collected and cells were detached using PBS-EDTA for protein quantification. Virus replication was assessed by monitoring either accumulation of influenza M1 protein in infected cells, or by titrating the viral infectious output present in supernatant. The titration of viral supernatants was performed by infecting MDCK cells plated in 48 well plates with serial dilutions of the viral supernatant. 20 hours later, cells were washed once with PBS, fixed directly in the plate with 100% methanol at −20°C for 10 minutes, washed once with PBS, and incubated for 30 minutes at room temperature in PBS 1% BSA. Infected cells were then revealed by immunofluorescent staining with a FITC-coupled anti-NP (#8257F from Millipore, at a 1/500th dilution in PBS) for 45 minutes at room temperature, followed by three PBS washes. Titer was computed by scoring the numbers of green cells under a fluorescence microscope.

## Results

### Balsamin Potently Inhibits HIV-1 Replication in T Cell Lines

**Figure 1:**
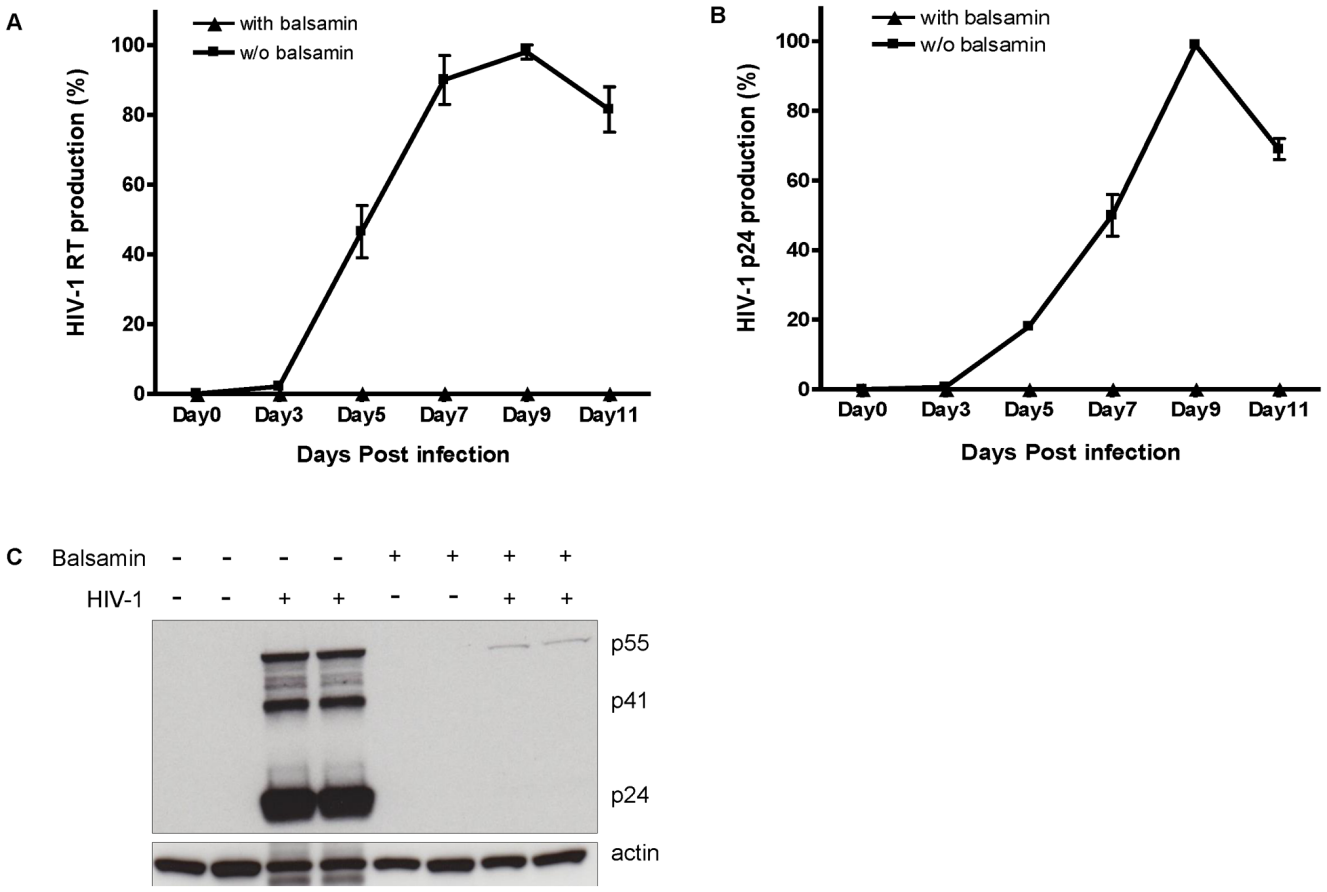
Balsamin potently inhibits HIV-1 replication in T cell lines. A. Jurkat cells were infected with HIV-1 at a moi of 0.01, in the absence or presence of 3.57 µM balsamin, and cell-free supernatant was collected at indicated days for monitoring HIV-1 production by RT assay or p24 capsid ELISA. B. The values obtained in the absence of balsamin were arbitrarily set as 100%. C. On the 11^th^ day, protein extracts were collected to analyze by Western blot the effect of balsamin on accumulation of HIV-1 p24, p41 and p55 proteins. Actin serves as a loading control. Error bars represent ±SD of two independent experiments performed in duplicate.

We first assessed the ability of balsamin to inhibit HIV replication in a viral growth curve assay in Jurkat T cells. Monitoring the release of viral proteins p24 and RT in the supernatant revealed that balsamin led to a dramatic decrease of viral replication. Balsamin (3.57 µM) addition indeed resulted in >99% inhibition of peak HIV-1 release in supernatant as measured by both RT assay (Figure 1A) and p24 ELISA (Figure 1B). At the end of the growth curve (11^th^ day), the amount of p24 in the supernatant of untreated cells was 6785 ng/ml of p24 versus only 20 ng/ml in balsamin-treated cells. To confirm these results, proteins were extracted from these cells on day 11, and analyzed by Western blotting. This again demonstrated that balsamin almost completely abolished accumulation of HIV-1 capsid protein in cytoplasm (Figure 1C).

**Figure 2:**
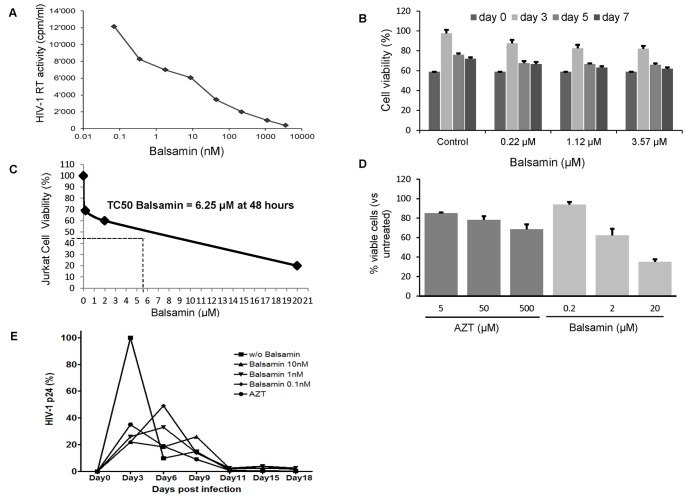
Determination of IC_50_ and cytotoxicity of balsamin. A. Dose-response curve of balsamin-mediated HIV-1 inhibition. Jurkat T cells were infected with HIV-1 at a moi of 0.1, in the absence or presence of indicated amounts of balsamin. Eight hours post-infection, cells were washed with PBS, and incubated further with relevant amounts of balsamin. Three days post-infection, cell free supernatant was harvested for assessing viral replication by RT assay. This allowed us to determine that the balsamin dose at which 50% of HIV-1 replication is 10.2 nM. The section of this figure is representative of four independent experiments. B. Monitoring in vitro cytotoxicity of balsamin. Different concentrations of balsamin (0.22, 1.12 and 3.57 µM) were applied on Jurkat T cells, and cellular viability was assessed by determining the percentage of viable cells using Trypan blue exclusion. Data represent ±SD of two independent experiments performed in duplicate. C. Determination of TC50 of balsamin in Jurkat cells. Jurkat cells were incubated for 48 hours with balsamin at 0.2 µM, 2 µM and 20 µM. At 48 hours cells were harvested for determination of cell viability by trypan blue assay and cell counting. One representative experiment out of two is shown. D. Jurkat cells were treated as above in parallel with AZT and stained for Annexin-V and 7-AAD. Viable cells (Annexin-V^−^/7-AAD^−^) were measured by FACS and plotted on a bar graph +/− SD (n = 2 in duplicate). E. Jurkat T cells were pre-treated with balsamin at sub-optimal concentrations (0.1 nM, 1 nM, 10 nM) for 3 hours. AZT (50 µM) was included as a positive control of inhibition. Cells were then infected with HIV-1 at a multiplicity of infection of 1.0 for the indicated days. Viral HIV-1 p24 concentration was measured by ELISA in harvested cell-free supernatants. A representative experiment out of two is shown with data expressed as percentage of HIV-1 p24 concentration measured in corresponding time points from supernatants of cells infected in the absence of balsamin.

In order to determine the IC_50_ of balsamin-mediated HIV-1 inhibition, we performed a dose-response curve on Jurkat cell line with increasing concentration of the protein. This indicated that balsamin exerts a dose-dependent inhibition of HIV-1 viral replication, with an IC_50_ value lying in the nanomolar range, namely 10.2 nM (Figure 2A). Finally, to exclude that balsamin antiviral effect is a consequence of a putative effect on cell health, we assessed the effect of increasing balsamin doses on Jurkat cells growth for 7 days (Figure 2B). In order to determine the TC50 of Balsamin, we performed a dose-response curve on Jurkat cell lines with increasing concentration of protein. Trypan blue exclusion counting method (Figure 2C) and a more quantitative Annexin V/7-AAD staining (Figure 2D) showed a TC50 of Balsamin at ∼6.25 µM in Jurkat cells after 48 hours treatment. A control experiment was performed using AZT and giving a TC50 of AZT at ∼329 µM (data not shown). Although the highest balsamin concentration affected the viability of Jurkat cells, no significant toxicity is observed at several doses at which balsamin already shows considerable antiviral activity. We conclude that balsamin potent HIV-1 inhibition is not due to a cytotoxic effect. As HIV-1 was shown to rapidly develop resistance to AZT via mutations in the reverse transcriptase viral genes [23], we aimed to investigate the emergence of drug-resistant escape mutants. We performed a dose-response curve on Jurkat cells in the presence and absence of increasing doses of protein over a period of 18 days upon HIV-1 infection. In the conditions used there was no emergence of resistance (Figure 2E). While this, however, does not preclude the possibility of resistance in other culture settings or indeed *in vivo*, these data demonstrate that the potent antiviral activity of balsamin is stable over time even at low concentrations.

### Balsamin Potently Inhibits HIV-1 Replication in Primary CD4^+^ T Cells

**Figure 3:**
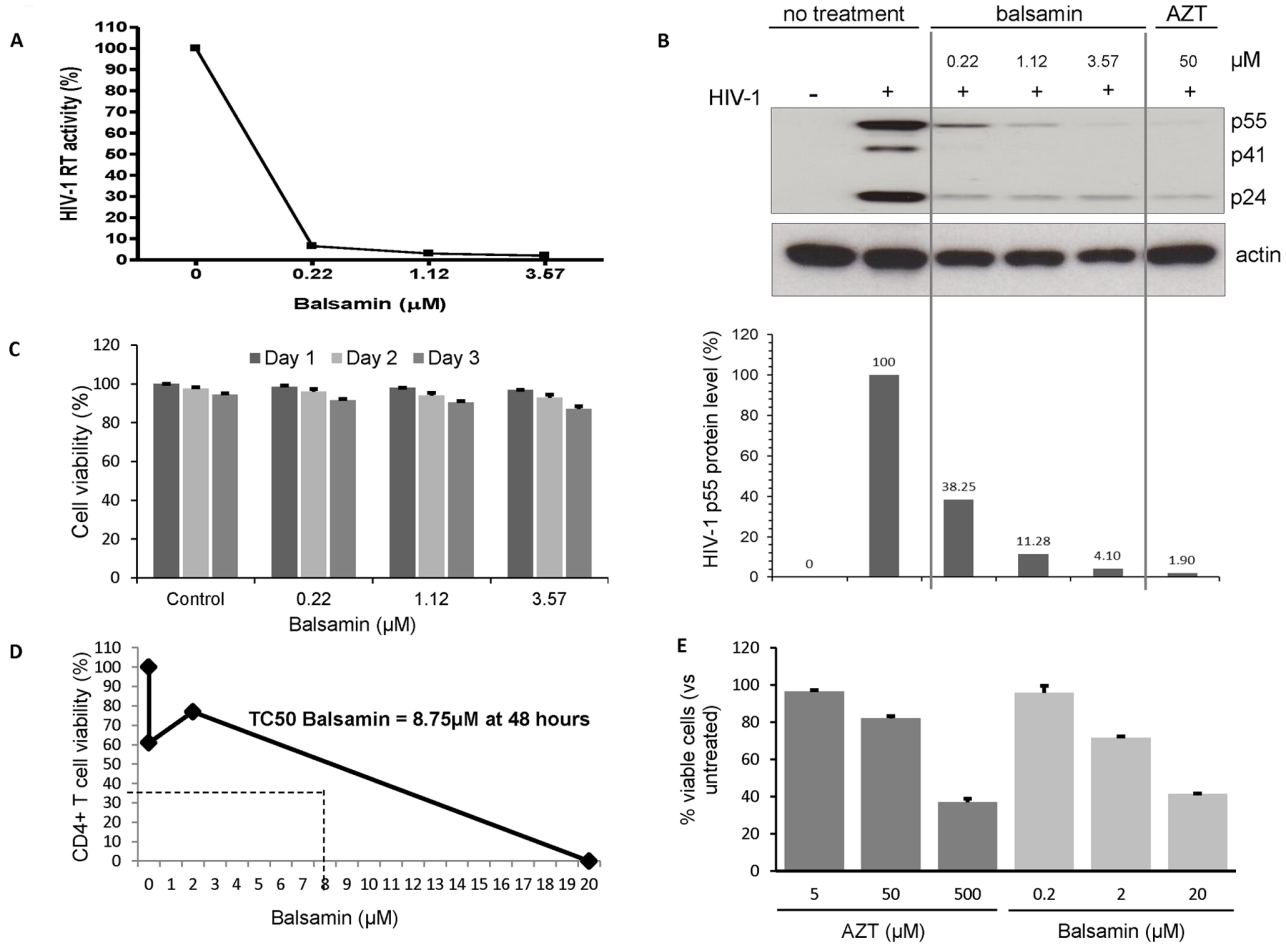
Balsamin potently inhibits HIV-1 replication in primary CD4^+^ T cells. A. Primary CD4^+^ T cells were infected with HIV-1 at a moi of 0.1, in the absence or presence of indicated amounts of balsamin. Eight hours post-infection, cells were washed with PBS, and incubated further with relevant amounts of balsamin. Three days post-infection, cell free supernatant was harvested for assessing viral replication by RT assay. The values obtained in the absence of balsamin were arbitrarily set as 100%. B. In parallel, cell lysates were collected and were immunoblotted for HIV-1 p24 (B, upper panel). Actin served as a loading control, and AZT was used as a positive control for the HIV-1 inhibition. The intracellular level of p55 was quantified and plotted (B, lower panel). The sections of this figure are representative of three donors. C. In parallel of this assay, putative cytotoxic effect of these different concentration of balsamin on primary CD4^+^ T cells were monitored by determining the percentage of viable cells using Trypan blue exclusion. Data are representative of three donors and experiments performed in duplicate (±SD). D. Primary CD4^+^ T cells were incubated for 48 hours with balsamin at 0.2 µM, 2 µM and 20 µM. At 48 hours cells were harvested for determination of cell viability by trypan blue assay and cell counting. One representative experiment out of two is shown. E. Primary CD4^+^ T cells were treated as above in parallel with AZT and stained for Annexin-V and 7-AAD. Viable cells (Annexin-V^−^/7-AAD^−^) were measured by FACS and plotted on a bar graph +/− SD (n = 2 in duplicate).

We next wanted to determine whether balsamin antiviral activity also extends to primary T cells. For that, we assessed the effect of increasing doses of balsamin on HIV-1 replication in these cells. Balsamin inhibited HIV-1 replication in a dose-dependent manner, as assessed by RT assay performed on the cells supernatant 3 days after infection (Figure 3A). Accordingly, intracellular HIV-1 p24 levels showed a similar dose-dependent decrease when monitored by Western blotting of balsamin-treated cells (Figure 3B). Importantly, measuring in parallel the cytotoxicity of balsamin on these cells showed an absence of deleterious effect on cell health, both over the range of balsamin doses used and over the period of the assay (Figure 3C). As in figure 2, we estimated the TC50 of balsamin in primary CD4^+^ T cells to be ∼8.75 µM by both Trypan blue exclusion counting method (Figure 3D) and Annexin V/7-AAD staining (Figure 3E). In comparison, TC50 of AZT in primary CD4^+^ T cells was ∼31 µM (data not shown).We conclude that balsamin potently inhibits HIV-1 replication in primary T cells, without affecting cellular viability.

### Investigating which viral Replication Step is Affected by Balsamin

**Figure 4:**
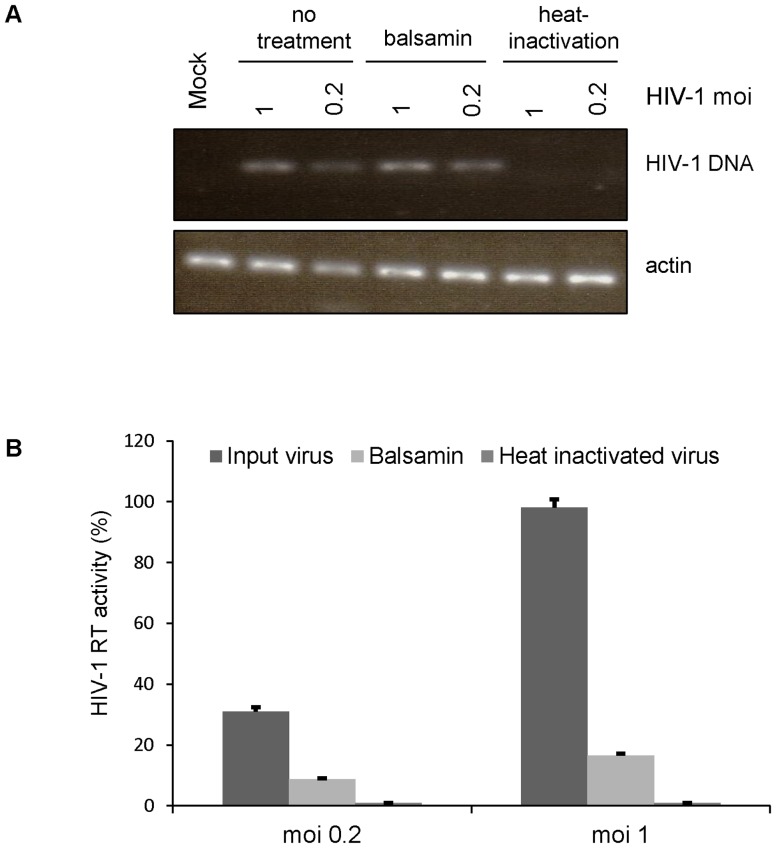
Balsamin does not inhibit early HIV-1 replication steps. A. Jurkat T cells were infected with DNase-treated HIV-1 with at a moi of 1 or 0.2. Eight hours post-infection, cells were washed with PBS and cultured in the presence or absence of 3.57 µM balsamin. Forty-eight hours later, cell-associated DNA was isolated and subjected to PCR with HIV-specific primers. Heat-inactivated virus served as a negative control. The section of this figure is representative of two independent experiments. B. In parallel, the effect of balsamin on HIV-1 replication was monitored by collecting the supernatant of infected cells and measuring viral release by RT assay. The values obtained in the absence of balsamin at moi 1 were set as 100%. Data are ±SD and representative of two independent experiments.

How RIP affect viral replication is a matter of debate [24]. To shed some light on how balsamin inhibits HIV-1 replication, we tried to delineate more precisely at which step of the viral replication cycle balsamin exerts its antiviral activity. We first infected Jurkat T cells with 2 different doses of HIV-1 and, forty-eight hours later, viral supernatant was collected and its content in HIV-1 RT was measured (Figure 4B), which showed that balsamin exerted a strong effect on viral replication as expected. In parallel, the accumulation of viral DNA in cells was monitored by PCR (Figure 4A). This showed that balsamin had no effect on the ability of the virus to reverse-transcribe its genome into DNA. Heat-inactivation of viral particles prior to infection served as negative control. This demonstrates that balsamin exerts its antiviral activity at a step later than reverse transcription but prior to viral particle production.

These results suggested that balsamin may exert its activity at the translation step of viral proteins, between reverse transcription of incoming viral genome and release of newly produced viral particles. In order to assess this possibility, we made use of a single-round HIV-1 system, where the virus is only capable for a single replication cycle. This system allows to precisely monitor the translation of viral proteins during one single round of replication, thereby avoiding the potentially confounding effect of subsequent rounds of replication. For that we used a HIV-1 construct that is able to perform only a single round of viral replication, due to a deletion of the envelope gene. The initial infection is performed owing to the incorporation of the VSVG envelope protein in the viral particles during the production of these viruses. During the initial round of infection, the HIV-1 proviral genome is integrated in the host DNA, which drives the normal transcription, translation, assembly and release of viral proteins. However, due to the absence of any viral glycoprotein, no infectious viruses are produced and therefore subsequent rounds of replications are fully abrogated [25].

**Figure 5:**
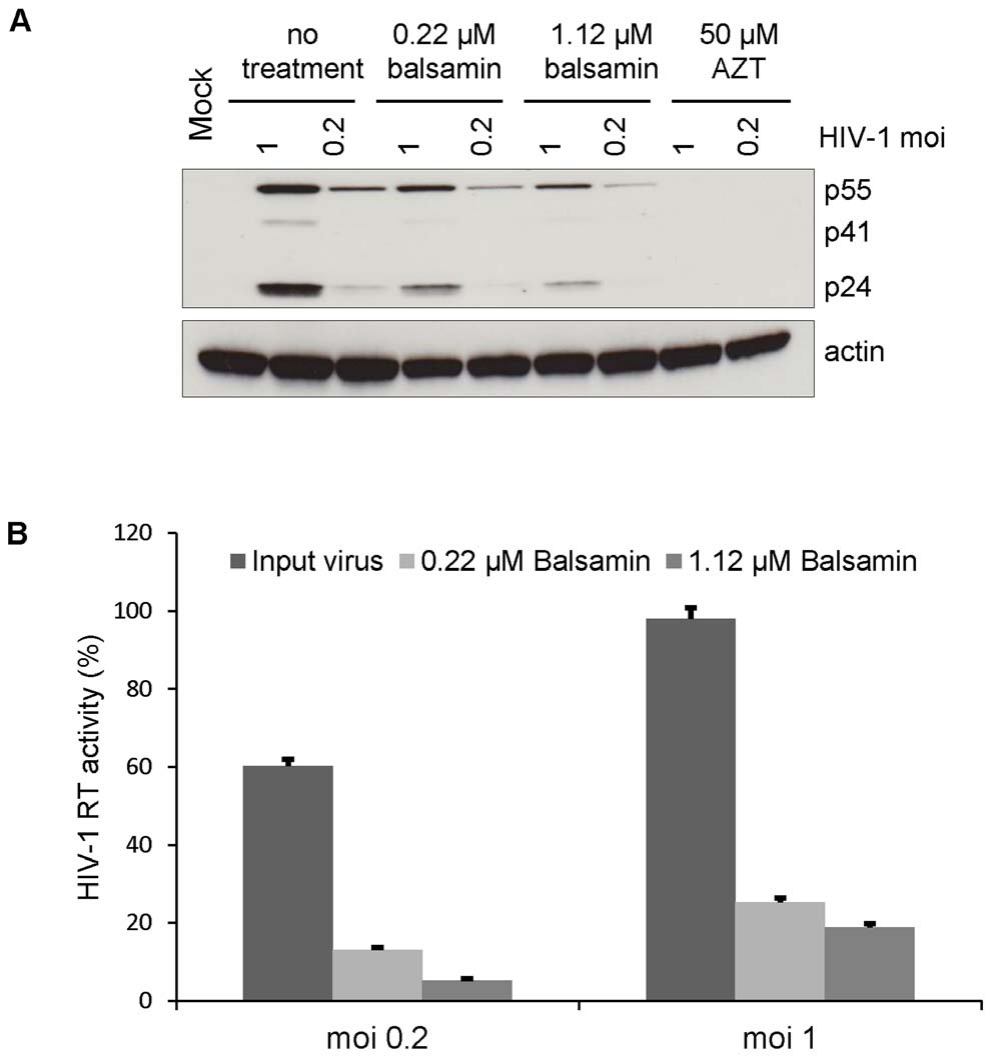
Balsamin inhibits HIV-1 at the viral proteins synthesis step. A. Primary CD4^+^ T cells were infected with DNase-treated single-round HIV-1 virus at a moi of 1 and 0.2, in the absence or presence of indicated doses of balsamin. Treatment with 50 µM AZT served as a positive control of HIV-1 inhibition. Forty-eight hours post-infection, cell lysates were prepared and subjected to Western blot analysis for HIV-1 p24 capsid and α-actin (as a loading control). The section of this figure is representative of two independent experiments. B. In parallel, cell-free supernatants were harvested from these cells 48 h of post-infection and viral replication was assessed by RT assay. The value obtained in the absence of balsamin at moi 1 was set as 100%. Data represent ±SD and is representative of two independent experiments performed in duplicate.

Primary CD4^+^ T cells were infected with two different viral doses of this single-round *env*-deleted HIV-1 virus. Forty-eight hours later, viral supernatant was collected and its content in HIV-1 RT was measured (Figure 5B), which showed that balsamin exerted a strong effect on viral replication even in this single round setting. Interestingly, Western blot analysis was performed in parallel on cellular extracts showed a comparable decrease of viral proteins accumulation in infected cells (Figure 5A). This shows that balsamin exerts its antiviral activity at or before the translation of viral proteins. We conclude from these results combined with results from Figure 4 that that balsamin exerts its activity between reverse transcription and the apparition of viral proteins, possibly at the translation step.

### Balsamin Potently Inhibits Influenza virus Replication

**Figure 6:**
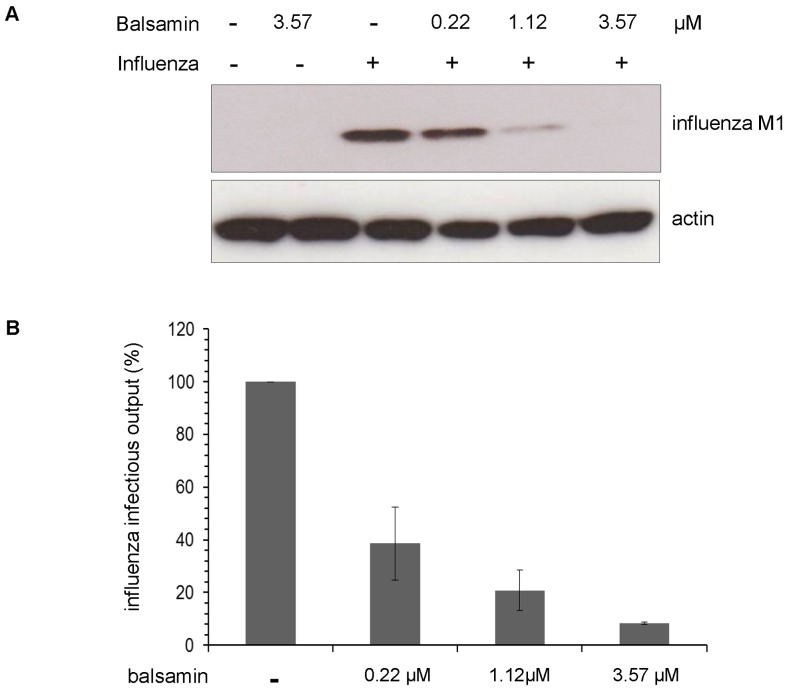
Balsamin potently inhibits influenza virus replication. A. A549 respiratory cells were infected with influenza virus PR8 strain cells at a moi of 1, in the absence or presence of indicated doses of balsamin, and then washed after overnight incubation. Twenty-four hours later, the impact of balsamin on influenza virus proteins accumulation in cells was monitored by preparing cell lysates and analyzing them by Western blotting for viral M1 matrix protein. Actin served as a loading control. The section of this figure is representative of two independent experiments. B. In parallel, the impact of balsamin on influenza virus replication was assessed by titrating the viral content of infected cells supernatant on MDCK cells. Data represent ±SD and representative of three independent experiments performed in duplicate.

In order to determine whether the antiviral activity of balsamin extends to other class of viruses, we addressed whether it was able to inhibit replication of the influenza virus, a RNA virus possessing a segmented negative strand genome. For that, A549 cells were infected with the PR8 strain of influenza virus in the presence of increasing concentration of balsamin. Twenty-four hours later, a Western blot analysis was performed on cell extracts (Figure 6A) to monitor the accumulation of the viral M1 protein. This demonstrated a potent dose-dependent inhibition of influenza replication by balsamin. Notably, visual inspection of the cells excluded that this decrease would be due to balsamin-induced cell suffering (data not shown). In parallel, viral supernatants were collected, and their content of infectious particles were determined by titration on MDCK cells (Figure 6B). This confirmed that balsamin treatment led to a potent dose-dependent reduction of production of new viral particles. Taken together, data suggest that balsamin antiviral activity is active against a broad range of viruses.

## Discussion

Ribosome-inactivating proteins (RIPs) are recognized to have various biological properties such as antimicrobial, abortifacient, anti-tumor, immunosuppressive and antiviral activity [5], [7]. Balsamin is a type I RIP recently isolated from *Momordica balsamina* seeds that has N-glycosidase activity and inhibition of protein synthesis in cell-free lysate [17]. The present study demonstrates that balsamin exerts potent anti-HIV activity, with an IC_50_ of approximately 10 nM. Balsamin indeed inhibits HIV-1 replication in T cell lines as well as primary CD4^+^ T cells, as scored by several assays, including growth curves and single-round assays, reaching >99% inhibition in growth curve assays. Importantly, a potent activity is observed also at doses where no significant toxicity to cells is observed.

The exact mechanism that RIPs use to inhibit viral replication is a matter of debate [24]. Notably it was proposed that three isoforms of PAP (PAP-I, PAP-II and PAP-III) from *Phytolacca americana* inhibited HIV-1 replication by causing dose-dependent depurination of HIV-1 RNA. In contrast to PAP isoforms, RTA (Toxic A chain of ricin from *Ricinus communis*) has no anti-HIV activity and fails to depurinate HIV-1 RNA. Intriguingly, both RTA and isoforms of PAP have ribosome-inactivating property, owing to highly conserved residues. Therefore anti-HIV activity of PAP isoforms may be due to its unique ability to depurinate HIV-1 RNA [26]. In another set of experiment, three major proteolytic fragments of MAP30 (*Momordica charantia*) and GAP31 (*Gelonium multiflorum*) were generated. These were N-terminal, central proteolytic fragment and C-terminal fragment. The central proteolytic fragment of MAP30 and GAP31 were able to inhibit HIV-1 p24 expression, prevent HIV-1 integration, and topologically relax supercoiled DNA but did not show any ribosome inactivation activity, whereas C-terminal region had ribosome inactivation activity. This suggested that anti-HIV activity of MAP30 and GAP31 was exerted independently of ribosome inactivating activity [27].

Finally, it was proposed that MAP30 (*Momordica charantia*), GAP31 (*Gelonium multiflorum*), luffin (*Luffa cylindrica*), saporin (*Saponaria officinalis*) and TCS (*Trichosanthes kirilowii*) affect viral replication by inhibiting HIV-1 integrase *in vitro* [28], [29]. In particular, TCS (*Trichosanthes kirilowii*) acted on HIV-1 LTR to specifically inhibit HIV-1 DNA integration [4]. In order to delineate at which replicative step balsamin exerts its antiviral action, we measured accumulation of HIV-1 reverse transcripts in infected cells. This showed that balsamin had no effect on this step, excluding an activity of this antiviral compound on viral entry and reverse transcription. In addition, we showed that the effect is already apparent shortly afterwards, at the level of accumulation of viral proteins in the cytoplasm, strongly suggesting that balsamin activity on protein translation underlies its antiviral activity, as was shown by for trichosanthin anti-HIV property [30]. Such a mechanism of action would also be consistent with the observed absence of viral escape mutants (Figure 2E), since a mutation rendering the viral replication independent of ribosomal translation is not possible. Accordingly, previous studies on MAP30 from *Momordica charantia* showed that its anti-HIV activity is exerted by decreasing p24 expression and viral RT activity [10]. Nevertheless, the exact mechanism underlying this antiviral activity is still to be fully unraveled. Additional mechanisms participating in antiviral potency may also be at play. In that respect, studies on trichosanthin, another type 1 ribosome-inactivating protein, showed that anti-HIV activity is not entirely dependent on ribosome-inactivating activity [31]. In addition to the inhibition of HIV-1, we here demonstrate a potent activity of balsamin against the influenza virus, another important human pathogen. This finding is reminiscent of the action of pokeweed antiviral protein against this pathogen [32]. This strongly suggests that balsamin has broad anti-viral activity, and therefore these observations may open new therapeutic avenues for a large scope treatment of viral infections.

### Conclusions

These studies demonstrate that balsamin expressed potent anti-HIV activity led to a decrease of viral replication. Balsamin effectively inhibited replication of HIV-1 in a dose-dependent manner in primary CD4^+^ T cells. Single-round infectivity assay also concluded that balsamin may exert its activity at that translation step of viral replication, between reverse transcription of incoming viral genome and release of newly produced viral particles. Finally, we demonstrate that balsamin antiviral activity is broad since it also impedes influenza virus replication. Based on these results, balsamin is a potential candidate that could be used as an antiviral agent for the treatment of viral infections.
